# Current Perspectives on Aptamers as Diagnostic Tools and Therapeutic Agents

**DOI:** 10.3390/pharmaceutics12070646

**Published:** 2020-07-09

**Authors:** Prabir Kumar Kulabhusan, Babar Hussain, Meral Yüce

**Affiliations:** 1 Department of Biotechnology, Gangadhar Meher University, Sambalpur 768001, India; kulabhusan007@gmail.com; 2 Faculty of Life Sciences, University of Central Punjab, Lahore 54000, Pakistan; bhussain@sabanciuniv.edu; 3 SUNUM Nanotechnology Research and Application Centre, Sabanci University, Istanbul 34956, Turkey

**Keywords:** aptamer drugs, aptamers in clinical trials, aptamers in diagnostics, aptamer–drug conjugates, aptamer-targeted delivery

## Abstract

Aptamers are synthetic single-stranded DNA or RNA sequences selected from combinatorial oligonucleotide libraries through the well-known in vitro selection and iteration process, SELEX. The last three decades have witnessed a sudden boom in aptamer research, owing to their unique characteristics, like high specificity and binding affinity, low immunogenicity and toxicity, and ease in synthesis with negligible batch-to-batch variation. Aptamers can specifically bind to the targets ranging from small molecules to complex structures, making them suitable for a myriad of diagnostic and therapeutic applications. In analytical scenarios, aptamers are used as molecular probes instead of antibodies. They have the potential in the detection of biomarkers, microorganisms, viral agents, environmental pollutants, or pathogens. For therapeutic purposes, aptamers can be further engineered with chemical stabilization and modification techniques, thus expanding their serum half-life and shelf life. A vast number of antagonistic aptamers or aptamer-based conjugates have been discovered so far through the in vitro selection procedure. However, the aptamers face several challenges for its successful clinical translation, and only particular aptamers have reached the marketplace so far. Aptamer research is still in a growing stage, and a deeper understanding of nucleic acid chemistry, target interaction, tissue distribution, and pharmacokinetics is required. In this review, we discussed aptamers in the current diagnostics and theranostics applications, while addressing the challenges associated with them. The report also sheds light on the implementation of aptamer conjugates for diagnostic purposes and, finally, the therapeutic aptamers under clinical investigation, challenges therein, and their future directions.

## 1. Introduction

Aptamers are a class of small single-stranded DNA or RNA oligonucleotides, with a three-dimensional structure enabling the target binding and specificity. The synthesis of aptamers is based on an in vitro evolution procedure, known as ‘Systematic Evolution of Ligands by EXponential Enrichment’ (SELEX) [1,2]. The strategy involves the iterative selection of high-affinity nucleic acid ligands towards a broad range of targets, including small molecules, proteins, peptides, toxins, whole cells, and tissues. Aptamer technology was developed in the 1990s, simultaneously, by two independent groups, Tuerk and Gold [3], and Ellington and Szostak [4]. The fundamental idea of the selection procedure is generally similar for both DNA and RNA oligonucleotides, and entails three critical steps. These are (i) incubation of the random oligonucleotide library with the selected target; (ii) partitioning or separation of bound oligonucleotides from the non-bound candidates, and (iii) recovery and amplification of the target-bound candidates for the next cycle. The process is iterated until the population is enriched with the aptamers bearing the desired function, sensitivity, and selectivity. Despite small molecular weight (5–30 kDa), aptamers can bind to their targets with high affinity, with the equilibrium dissociation constants (K_D_) ranging from a low nanomolar to a high picomolar range. The specificity of the aptamers and other unique attributes such as ease in chemical synthesis, tunable backbone modification, low immunogenicity, and excellent stability [5,6], allow aptamer oligonucleotides to offer multiple advantages over antibodies, in medical diagnostic, bio-sensing, and bio-imaging platforms. Antibodies are usually bound to proteins for diagnostic procedures, such as enzyme-linked immunosorbent assay (ELISA). However, aptamers can bind to a variety of targets such as bacteria/pathogens [7,8], proteins [9], toxins [10], viruses [11], live cancer cells [12], and tissues [13]. They are stable in a range of ionic conditions, temperature, and pH. Additionally, in vitro selection of aptamers helps to avoid complications arising from the use of animals. Aptamers can be chemically modified with functional groups or chemicals or nanoparticles that make them a perfect material for diagnostic applications [5,8]. The small size of the aptamer oligonucleotides ensures their penetration into tumor cells, as compared to antibodiesAs we reviewed earlier [14], aptamers could be conjugated with non-coding RNAs, drugs, nanoparticles, and proteins for sensing, imaging, and targeted drug delivery purposes.

Apart from the traditional aptamers, continuous efforts are being taken to develop aptamers for a wide range of clinically relevant diseases, by modifying the selection procedures, types of targets, and the synthetic chemistries. In the conventional strategy, usually, 10–12 rounds of selection rounds are required to obtain the enriched specific DNA/RNA aptamer pools, after which they are validated using standard characterization techniques, such as pull-down assays, gel electrophoresis, or bilayer interferometry. Nowadays, several other analytical approaches, such as capillary electrophoresis [15], particle display technology [16], and microfluidic chips [17], have been introduced along with the SELEX process, to improve the efficiency of the selection procedure. Moreover, it has become possible to add multiple functional moieties onto the aptamers through automated modular synthesis, computational technologies, and phosphoramidite chemistry [18]. For example, Meng Liu et al. (2019) reported the selection of two high affinity DNA aptamers from the circular libraries [19], against the glutamate dehydrogenase (GDH) of *Clostridium difficile*. These two aptamers were recognized by different epitopes of the GDH antigen, suggesting the advantages of aptamer selection from a circular DNA library. Another approach is the Microfluidic SELEX (M-SELEX) method reported by Lou et al. (2009). The researchers implemented a design that enabled beads that isolated high-affinity aptamers, efficiently and rapidly [20]. The significant advantages of M-SELEX are that it can select aptamers for any target, which can bound to the bead, and once the target is immobilized on the magnetic beads, the separation parameters such as magnetic actuation and flow rates do not require additional optimization. Furthermore, the M-SELEX method utilizes higher molar ratios of the initial DNA library and the target proteins, which leads to higher efficiency in selection. As mentioned earlier, the conventional SELEX includes 10–12 iterated rounds of nucleic acid amplification. This process might become time-consuming and prone to failure because there is a high chance that PCR amplifies the non-binders and fails to select the binder sequence in 70% of attempts [21]. To address the issue, Liu et al. (2019) reported a one-step Ideal-Filter capillary Electrophoresis (IFCE) device, wherein the binders and the non-binders move in the opposite direction, under the influence of the electric field [15]. The result suggests that the efficiency of the IFCE-based selection method is significantly higher than that of conventional SELEX. This method is fast, robust, and can be used in different types of oligonucleotide libraries. A similar line of advancements in the SELEX procedure was expended to tissue SELEX [22], in vivo SELEX [23], and 3D-cell SELEX [24]. Aptamers were previously vulnerable to nuclease digestion in the body that was enhanced by changing the aptamers chemically, to increase the in vivo stability and half-life. This can be done by replacing the 2′-OH with several functional groups like amino (NH_2_), *O*-methyl (OCH_3_), or fluoro (F) groups. Another approach is to utilize inverted dT residue for capping the 3′ end of oligonucleotides [25,26], to improve the stability of aptamers. The recently developed SELEX methods shortened the time from weeks to hours and from multiple rounds to even a single round. The next-generation sequencing platforms, computational simulations, and high-throughput screening methods were integrated into the SELEX procedures to make the technique more dynamic and user friendly. These technologies provided significant insights during selection procedures, easing up the scale-up operations, and delivering highly efficient aptamers at a low cost.

The DNA aptamers as targeting ligands to transport therapeutic agents specifically to tumors were of significant interest in recent years. Li et al. (2018) utilized DNA origami to construct nanorobots functionalized with DNA aptamers (AS1411) that bind to nucleolin, a surface receptor explicitly expressed on cancer cells [27]. The nanorobot opened in the presence of the aptamer to expose the therapeutic cargo, i.e., thrombin, which caused an inhibition of tumor growth. Futami et al. (2019) utilized the artificial (unnatural) base pair in SELEX (cell-ExSELEX) to generate high affinity and versatile DNA aptamers towards cancer cells [28]. Similarly, Matsunanga, et al. (2017) used one unnatural hydrophobic base, 7-(2-thienyl) imidazo [4,5-b] pyridine (Ds) to increase the complexity of the library, for the selection of aptamer targeting to the A1-domain of von Willebrand factor (vWF). The Ds containing aptamer displayed significantly efficient affinity (K_D_ = 75 pM), as compared to any aptamer containing unnatural nucleic acid bases [29]. Furthermore, several types of such modification strategies were adopted to increase the nuclease stability, affinity, and specificity of the aptamers. SomaLogic Inc. developed Slow Off -rate modified aptamer (SOMAmers), which contains modified nucleic acid bases such as 5-(N-benzyl carboxamide)-2-deoxyuridine (Bn-dU) or 5-[N-(1-naphthylmethyl) carboxamide]-2-deoxyuridine (Nap-dU). These modified bases commonly used to select aptamers against small molecules and challenging protein targets, such as toxins. The SOMAmers display significantly higher affinity in the nanomolar to picomolar range, towards its target. Similarly, Noxxon pharma generated nuclease resistant aptamers using L-ribose nucleotides instead of natural D-ribose [30]. This novel class of aptamers is known as ‘Spiegelmers’ or mirror-image aptamers [31,32]. Other strategies, such as phosphodiester linkage modifications [33], end-capping [34], and truncation tailored SELEX [35] have assisted in improving the therapeutics approach of the designed aptamers. Another remarkable progress is the development of bifunctional aptamers, wherein two or more types of aptamers are connected and utilized together for therapeutic purposes. Macdonald et al. (2017) fused an aptamer specific for the epithelial cell adhesion molecule (EPCAM) and the transferrin receptor (TfR) [36]. The study showed that the fusion of the aptamer improves the binding affinity, while maintaining the same specificity. The fused aptamer could cross the blood–brain barrier (BBB), and could be used for brain disorder therapeutics. The same research group also demonstrated the doxorubicin (DOX) loaded bifunctional aptamer (EPCAM-TfR), where the DOX was intercalated into the double-stranded aptamer arm. The drug-loaded aptamer vehicle could transcytose the endothelial cells of the BBB and deliver the payload for the treatment of EPCAM positive brain metastasis cells [37]. Considering the challenges, the novel modification strategies that are coming up and the need to draw them together for a better understanding of aptamer technology, we summarized the most recent developments in aptamer as diagnostic and therapeutic purposes, throwing light on biomarker discovery and clinical evaluation of aptamers that are already developed and marketed, and those in the pipeline.

## 2. Aptamers in Cancer Diagnosis

Early diagnosis of cancer before any symptoms appear is crucial for the treatment of the disease. The computed tomography (CT), magnetic resonance imaging (MRI), immunohistochemistry, cancer biomarker assays in serum (e.g., ELISA), and flow cytometry are commonly employed for the diagnosis of cancer. However, low concentrations of cancer biomarkers in serum or plasma, mixed with other proteins make the early cancer diagnosis quite tricky. Additionally, these methods could be labor-intensive and time-consuming [25]. Therefore, due to their higher sensitivity i.e., the ability to detect quite low quantities of their targets [7,8], aptamers have become an emerging and promising tool for cancer diagnosis and imaging. Aptamers were used to identify as low as 10 cancer cells [38]. Furthermore, aptamers can recognize several cancer metabolites, differentiating cells, molecules affecting tumor behavior, or cancer biomarkers. One of the most used detection systems for cancer diagnosis is aptamer-nanoparticle (Apt-NP) conjugates. These conjugates can be used to detect cancer cells from the complex body fluids such as serum and blood. Tumor cell-specific aptamers can be quickly immobilized on the surface of nanoparticles, such as a gold nanoparticle, magnetic nanoparticles, and quantum dots or carbon-based nanoparticles (mainly graphene and its derivatives). The aptamers perform the highly sensitive and selective detection of cancer cells, while the nanoparticles protect aptamers against nuclease activity. For example, Borghei et al. (2016) developed a highly sensitive and simple colorimetric method to detect MCF-7 breast cancer cells, using AS 1411 aptamer nucleotide-gold nanoparticle (AuNP) conjugates. The cancer cells captured the aptamers due to its affinity with cells’ nucleolin receptors. The binding of AS 1411 aptamer to the breast cancer cells resulted in its removal from the solution [38]. In this way, no aptamers were left in the solution to recombine with the complementary ssDNA-AuNP probes, so the solution had a red color. In the absence of targeted cancer cells, however, aptamers and ssDNA-AuNP conjugates coexisted in solution, forming a distinct purple color. This method detected breast cancer cells at a concentration as low as 10 MCF-7 cells. An alternate aptamer selection strategy was reported recently that identified aptamers for the diagnosis of hormone-refractory prostate cancer in an orthotopic xenograft mouse model [39]. For this purpose, random DNA libraries were employed for identifying aptamers that specifically targeted androgen-independent prostate tumors in living mice. Multiple tumor-targeting aptamers were identified through recurrent SELEX cycles, and the D3P-21 aptamer had high affinity and specificity for in vivo diagnosis of prostate tumors. The aptamer–tumor interaction depended exclusively upon the attachment of polyethylene glycol (PEG) moiety at the 5′-end of the oligonucleotide. An aptamer-fluorescent silica nanoparticle (FSNPs) conjugate system was developed by Tan et al., (2016) for the detection of blood cancer or leukemia cells. Amine-labeled Sgc8 aptamers were conjugated to carboxyl-modified FSNPs for highly specific and sensitive detection of leukemia cells [40]. Another highly selective and sensitive biosensor for early identification of T-cell leukemia was reported using the same Sgc8 aptamer, [41]. A truncated Sgc8 aptamer was conjugated to AuNP-coated magnetic Fe_3_O_4_ NPs (Apt-GMNPs). Additionally, the stem of the aptamer hairpin was intercalated with ethidium bromide (EB), and the presence of cancer cells disrupted the hairpin and released the EB, thus reducing the electrochemical signal. This approach detected the leukemia cells between 10 to 1 × 10^6^ cells per ml of the tested sample. Similarly, Hu et al., (2017) developed an aptamer-fluorescent silica NPs conjugate in which the target-specific aptamers were not directly attached but interacted via biotin–streptavidin interaction. The liver cancer cells, HepG2, were incubated with biotin-linked TLS11a (Bio-TLS11a) aptamer [42]. The highly specific and robust biotin-streptavidin association was the basis of the detection of cancer cells, through the incubation of Bio-TLS11a and streptavidin-linked FSNPs. This method resulted in a specific and sensitive diagnosis of hepatoma cancer cells. In addition to FSNPs, other fluorescent NPs, such as quantum dots (QDs) were utilized in the diagnosis of cancer tumors, for example, AS1411 and TTA-1 aptamer-conjugated with QDs were used for multiplex detection of two cancer biomarkers (nucleolin and tenascin-C) in three different cancer cell lines [43]. Similarly, aptamer-QD conjugates were used to identify lymphoblastic leukemia cells (CCRF-CEM) through the use of cell surface receptor protein tyrosine kinase 7 (PTK7) specific aptamers, and cancer cell imaging was obtained in the presence of cancer cells through QD-based fluorescent cellular imaging [44]. In another study, A32 aptamer-QD conjugates were used for fluorescence-guided surgery for glioma (brain and spinal cord) cancer cells [45]. It was found that A32 aptamers presented a strong affinity towards the epidermal growth factor receptor variant III found on the surface of glioma cancer cells, and it was measured in terms of fluorescence. In a novel approach, the J3 aptamer was conjugated with the Cy5-fluorescent group (J3-Cy5) for the detection and bio-imaging of metastatic cancer cells [46]. The J3-Cy5 conjugates identified the colorectal (colon/bowl) carcinoma metastasis with a 73.9% efficiency, thus showing the potential of this system in diagnosis, and further opened a room for further improvement. 

Ovarian cancer is one of the most lethal gynecologic tumors that are not easy to be detected at an early stage, which contributes to a high mortality rate. In a clinical study of ovarian cancer patients, the affinity of R13 aptamer with cancer cells was studied through flow cytometry analysis [47]. Additionally, the stability of R13 aptamer was also analyzed in an ovarian cancer patient’s blood serum. The verification of aptamer binding to cancer cells was performed through confocal microscope imaging, using 80 ovarian cancer tissues and ten normal ovary tissues as a control. The affinity of R13 to cancer cells was further confirmed in NOD/SCID tumor models of mice. The R13 aptamer showed a high binding affinity with several ovarian cancer cell lines (Caov3, HO8910, A2780, and SKOV3) with K_D_ values in the nanomolar range (158 ± 28.22, 47.48 ± 7.15, 29.24 ± 8.55, 37.87 ± 4.93 nM, respectively). In another study conducted by Pan et al. (2018), six aptamers with high affinity against gastric cancer biomarkers (CEA, CA72-4, and CA50) were identified from a randomized 30mer RNA library. The predicted secondary structures of aptamers for these biomarkers had a significant structural similarity. Fluorescence spectroscopy analysis found the dissociation constants (K_D_) of CEA, CA72-4, and CA50 gastric cancer biomarkers to be 16.5-156 nM, 52.7-71.2 nM, and 30.7-38.0 nM, respectively [48]. The positive fluorescent signal from the immunostaining of gastric adenocarcinoma (AGS cell line) with the CEA aptamer probe further adds to the potential of the RNA aptamer in gastric cancer bio-sensing and bio-imaging. In 2019, another group reported four highly specific aptamers (Seq-3, Seq-6, Seq-19, and Seq-54) selected through the whole-serum subtractive SELEX method that presented the significant binding ability to gastric cancer serum. The K_D_ of these aptamers were 128 ± 26.3 nM, 149 ± 23.6 nM, 232 ± 44.2 nM, 202 ± 25.6 nM, respectively [12]. These oligonucleotides were applied for developing a qPCR protocol for early gastric cancer detection, due to the high target-specificity. These aptamers were used for detecting APOA1, Importin subunit alpha-1, PARD3, and APOA4 gastric cancer biomarkers; where Importin subunit alpha-1 is the strongest candidate to be used for further gastric cancer diagnosis. Similarly, colorectal cancer is one of the most common cancer, and there are no effective treatment measures are yet available. Hashkavayi et al. (2017) developed an electrochemical diagnostic platform by employing aptamers on an SBA-15-3-aminopropyltriethoxysilane (SBA-15-pr-NH2), and AuNPs modified graphite screen-printed electrode (GSPE) for the detection of CT26 cancer cells. The diagnosis was highly specific and did not show any cross-reactivity with other cancer cells such as the AGS, VERO, and SKOV-3 cell lines. The results obtained from the cyclic voltammetry and impedance spectroscopy suggest that the developed diagnostic had a limit of detection (LOD) of 2 cells/mL [49]. The use of aptamers for cancer detection was combined with clinical devices, such as ultrasound and MRI. For example, fluorescein-labeled aptamers conjugated with an ultrasound-propelled gold nanowire motors (FAM-AIB1-apt) coated with graphene-oxide (C_140_H_42_O_20_) was used for qualitative diagnosis of overexpressed AIB1 oncoproteins in breast cancer cells (MCF-7). The nanomotor movement in the ultrasound field ensured the faster uptake and binding of the aptamers with the target and results in fluorescence [50]. The propulsion of the nanomotors conjugated with aptamer significantly increased the fluorescence intensity, in comparison to the static conditions. Similarly, the interaction between Sgc8 aptamer and T-acute leukemia cells (CCRF-CEM) at variable flow conditions in microfluidic devices was also investigated. Additionally, the effects of device shapes and flow rates on aptamer–leukemia cell affinity were studied [51]. The MRI technology utilizes the protons’ behavior in magnetic fields to develop 3D images of body parts and biological systems for bio-imaging and diagnosis purposes. Efforts were made to improve the diagnosis capacity of MRI for various disease conditions, including the cancer diagnosis. For example, TLS11a aptamer was conjugated with Fe_3_O_4_ NPs that had a fluorescent SiO_2_ shell (MFS) for the detection and bio-imaging of HepG2, liver cancer cells. The fluorescence and MRI showed the specific uptake of aptamers by HepG2 cells, which was not observed for other cancer cell types (4T1, MCF-7, SGC-7901). This was also confirmed by MRI imaging of mice with liver tumors, while nanoprobes showed excellent biocompatibility and low toxicity [52], depicting the diagnostic potential of the system. Similarly, AS1411 aptamer-conjugated with Fe_3_O_4_-Au nanoparticles were used for the imaging and diagnosis of breast cancer cells expressing nucleolin. The MRI imaging found a significant increase in the signal intensity from the interaction of aptamer-NPs conjugates and different breast cancer cells [53]. This nanoprobe bound the 4T1 breast cancer cells with a higher specificity and affinity, and could be used as an MRI contrast agent.

The ability of aptamers to specially bind to living cells and tissues, their non-toxicity, high stability as compared to protein-based affinity reagents, and their ability to bind to several kinds of nanoparticles, makes them a powerful tool for targeted drug delivery to cancer cells. Recently, an AS1411 aptamer was used to deliver C8, a G-quadruplex ligand based on acridine, to cervical cancer cells or HeLa [54], highlighting its pharmaceutical potential. Similarly, aptamers were used to provide large RNA payloads (175–250 nt) to several human B cell cancer lines. Aptamers conjugated with fluorogenic RNA reporter specifically bound with leukemic B cell lines, thus caused fluorescence that lasted for ≥2 h [55], showing the potential use of the system for drug delivery to cancer cells. In another study, LC09 aptamer was used to specifically target the vascular endothelial growth factor A (VEGFA) of osteosarcoma cancer cells, by functionalizing LC09 aptamer with a PEG-PEI-Cholesterol (PPC) lipopolymer that encapsulated Cas9 and CRISPR/Cas9 plasmids coding for VEGFA gRNA. The specific binding of LC09 ensured the delivery of CRISPR/Cas9 to lung metastasis and orthotopic osteosarcoma [56], thus causing efficient VEGFA genome editing in cancer cells and inhibiting both types of tumors. Finally, a 59-base DNA aptamer, AB3, was used to target the immature laminin receptor protein (OFA/iLRP) in the acute myeloid leukemia (AML) cancer cells. The aptamer was functionalized to deliver doxorubicin (Dox) drug molecules to AML cells. The Aptamer showed a K_D_ of 101 nM with OFA/iLRP, but did not bind to albumin, ovalbumin trypsin, and OFA/iLRP-negative control cells [57]. The aptamer-doxorubicin (Apt-Dox) conjugate was made by placing the doxorubicin into the aptamer DNA structure, and it delivered the Dox to OFA/iLRP-positive AML tumor cells with high selectivity and sensitivity, and thus efficiently destroyed the AML cells. In conclusion, aptamers and aptamer–nanoparticle conjugates have emerged as a robust tool for the diagnosis of almost all types of cancers and are rapidly being applied in early clinical studies for rapid and reliable diagnosis and bio-imaging purposes. A list of these aptamers applied for cancer detection was given in Table 1.

## 3. Aptamers in Infectious Disease Diagnosis

Recently, due to globalization and the world being interconnected, infectious diseases spread very quickly and have created a serious public health concern. The healthcare-associated diseases cause severe socio-economic burden ($150 billion per year in the US) to the people [59,60]. To combat infectious diseases, early detection of infections is vital in its treatment and management. The conventional diagnosis methods, such as immunological methods like ELISA, polymerase chain reaction (PCR), and pathogen isolation, culturing for growth and microscopic identification, require around 24 h or more extended periods. Additionally, protein-based antibody methods are outclassed by DNA aptamers in several ways; for instance, aptamer biosensors do not require a lot of reagents and instruments like PCR and ELISA. Furthermore, aptamers are more stable as compared to protein-based affinity agents and give robust and specific results, while false positive and negative results are also prevalent in ELISA [2,10]. Nowadays, several aptamers are routinely being used for the diagnosis of infectious disease. For example, Suh et al. (2018) developed a DNA aptamer-based sandwich assay to capture and detect *L. monocytogenes* [61]. Immunomagnetic beads were utilized to capture the pathogen, followed by highly specific detection by the aptamer. The quantitative detection was performed by qPCR-based amplification of cell-bound aptamer, and as low as 2.5 CFU of bacteria in 500 uL buffer were detected.

Fluorescence resonance energy transfer (FRET) is a powerful phenomenon for studying the molecular interactions that were extensively used in biosensing and diagnosis applications [62]. A FRET aptasensor was recently reported [63] that utilized up-conversion nanoparticles (UCNPs) as donors, and AuNPs as acceptors for ultrasensitive and rapid diagnosis of *Escherichia coli* ATCC 8739 bacteria target In this system, aptamers were conjugated with AuNPs, and their corresponding complementary DNA (cDNA) was conjugated with UCNPs. The spectral overlap between emission and absorption of UCNPs and AuNPs, respectively, resulted in the FRET phenomenon when aptamer and cDNA hybridized, thus, causing up-conversion fluorescence quenching. The aptamers had more affinity with target bacteria, as compared to cDNA, thus, forming a 3D structure that led to the dissociation of UCNPs-cDNA from AuNPs-aptamers conjugate; this resulted in the recovery of up-conversion based fluorescence. This UCNPs-FRET aptasensor successfully diagnosed *E. coli* ATCC 8739 in the detection range and the limit of 5–106 cfu/mL and 3 CFU/ml, respectively. The FRET was used to detect *E. coli* in water and milk samples within 20 min. This highlights the power of FRET for a rapid and accurate diagnosis of pathogens and their potential for bio-imaging of infectious pathogens.

Similarly, AU1 and AD1 aptamers, selected through SELEX, were used for the detection of profoundly invasive *Candidiasis* (infection caused by candida fungus). Both aptamers showed high affinity and specificity with (1→3)-β-D-glucans present in the cell wall of the fungus, *Candida albicans.* The K_D_ values for AD1 and AU1 targets were predicted as 79.76 nM and 103.7 nM for (1→3)-β-D-glucans, but the binding domain of (1→3)-β-D-glucans was not the same for both aptamers [38]. The diagnosis of (1→3)-β-D-glucans in blood samples in patients with *Candida albicans* infection was also performed by a double-aptamer sandwich enzyme-linked oligonucleotide assay (ELONA) that showed 91.94% and 92.31% specificity and sensitivity, respectively.

**Figure 1:**
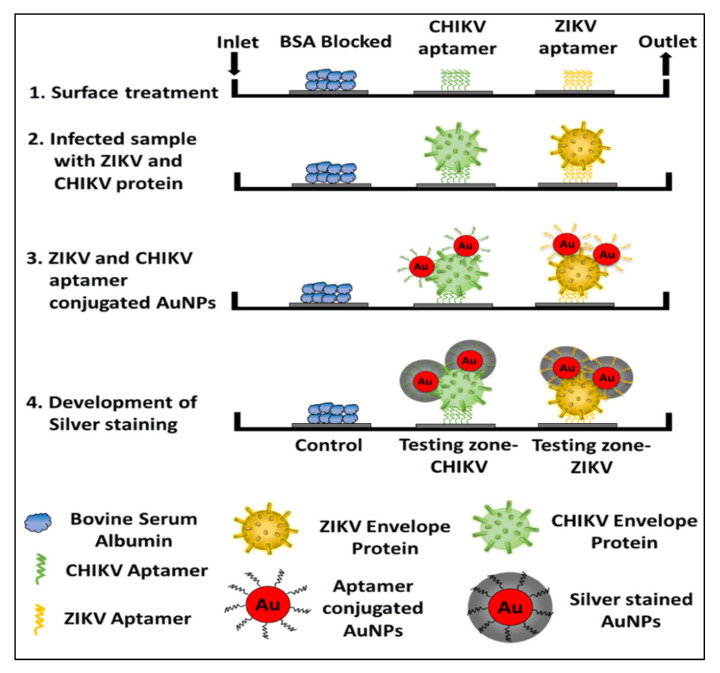
The schematic representation of a sandwich-type aptasensor platform for the detection of multiple viral proteins from various viruses, for example, the CHIKV and Zika virus. The image is reused with permission from [66] https://pubs.acs.org/doi/10.1021/acsomega.8b03277. Further permissions related to the material excerpted should be directed to the ACS.

In addition to bacteria and fungi, the aptamers were utilized for the diagnosis of a wide range of infectious viruses. For example, a novel aptamer S15 was reported with high affinity and specificity for the envelope protein domain III (ED3) of dengue virus 2 (DENV)). Circular dichroism studies found that the aptamer formed a parallel quadruplex on the virus. Both the quadruplex structure and a 5’-end sequence were required for the binding of S15, and it bound to an extremely conserved loop between the βB and βA strands of ED3 [64]. Additionally, S15 was able to neutralize the infections caused by all four DENV serotypes. Similarly, DNA aptamers and their truncated sequences obtained through the SELEX technology showed a high affinity and selectivity against type A influenza viruses (H3N2 and inactive H1N1 viruses), with a low nanomolar range of K_D_ [11]. These truncated sequences for H1N1 in ELONA showed a detection limit (LOD) of 0.3 ng/uL. In another study, an RNA aptamer, 39SGP1A, functionalized with 2’ fluoropyrimidine (2’FY) was developed, which was explicitly bound to a soluble glycoprotein (sGP) of the Ebola virus (EBOV). The motif of 39SGP1A aptamer was found to be a novel polypyrimidine-rich sequence that had a role in the recognition of sGP [65]. The aptamer was useful for the diagnosis of two Ebola virus species, Ebola virus (EBOV) and Sudan virus (SUDV, by binding to a conserved epitope present in both species. Recently, Saraf et al. (2019) developed a device for multiplexed diagnosis of chikungunya and Zika viruses through binding with their envelope proteins. Aptamers with high selectivity towards viruses were attached to the micro-sized pillars of a microfluidic channel. The detection took place inside the microfluidic channel and the pillars aided in enhancing the surface of the sensing area [66]. The device works with the interaction of a protein-mediated sandwich with an aptamer-AuNP conjugate. The subsequent introduction of a silver reagent and its deposition on the AuNPs surface, created a gray contrast in the testing zone. In this way, colorimetric aptasensor can precisely diagnose the chikungunya and Zika envelope proteins in calf blood samples (100 pM) and phosphate-buffered saline (1 pM). The schematic of the detailed colorimetric multiplex aptasensor is represented in Figure 1 [66].

In conclusion, aptamer-based bioassays were successfully utilized for the rapid detection of notorious infectious pathogens, such as Dengue virus 2, H3N2 and H1N1 influenza viruses, and the Ebola virus. Some of these infectious viruses have an epidemic and even pandemic outbreak potential. Therefore, the rapid, sensitive, and reliable detection of infectious pathogens through aptamers and aptamer-nanoparticle conjugates-based FRET system could potentially address the problem of slow testing and false negative/positive in a pandemic situation, such as the novel coronavirus (Covid-19), as FRET can give reliable results in just 20 min [63]. Additionally, the integration of aptamer-based diagnosis with medical devices such as MRI has been demonstrated [53], which shows the potential of aptamer use in diagnostic labs; but further research is needed to integrate aptamers in the applied diagnosis of infectious diseases. A summary of the aptamers used in contagious pathogen detection is presented in Table 2.

## 4. Aptamers as Therapeutic Agents

**Figure 2:**
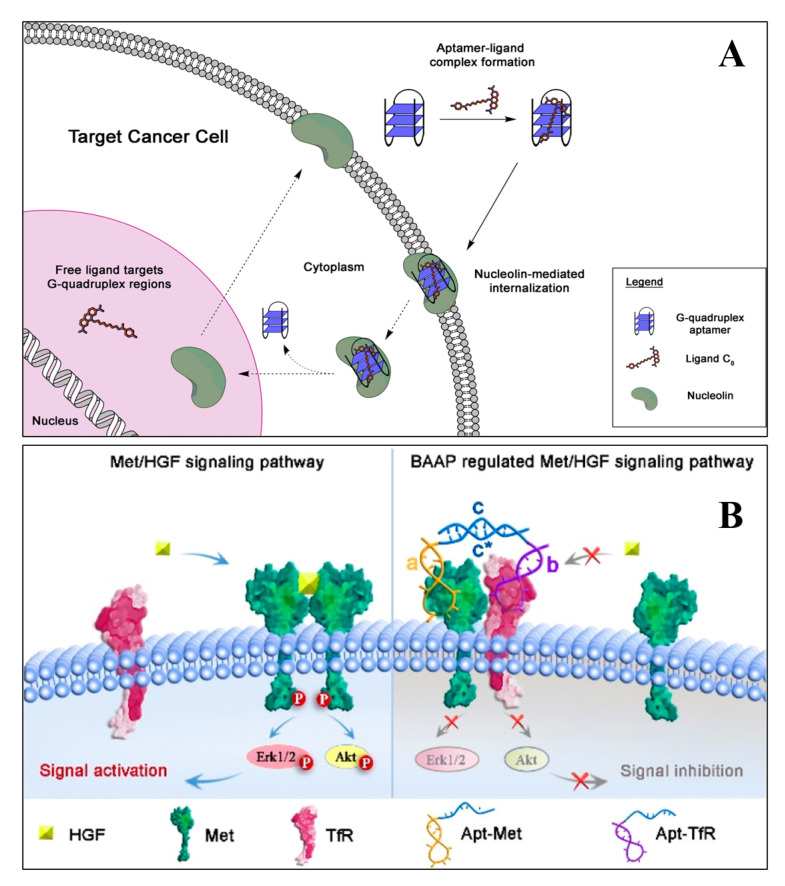
The schematic demonstrates the hypothesis of AS1411 aptamer and its ligand C8 into cancer cells (**A**) [54]. (**B**) The illustration of bispecific aptamers induced artificial protein pairing to regulate Met receptor pathway and its downstream signaling (**B**) [73].

Aptamers with high specificity and affinity for both protein and non-protein targets are widely used in therapeutic applications. The aptamers commonly used for therapeutic purposes are either selected by in vitro selection using a purified protein/receptor or through in vivo selection, using suitable model systems [67]. Therapeutic aptamers can be used as agonists, antagonists, and inhibitors or as targeting ligands [68]. Many of the aptamers in clinical trials are either antagonistic or inhibitory. These aptamers block or inhibit the interaction of disease-related targets through protein–protein interaction or through protein–receptor–ligand interaction. However, agonist aptamers activate the functions of target receptors and also serve as carriers to deliver the cargos to the target cells or the tissues [69]. Numerous research groups have developed aptamers for therapeutic purposes for various diseases, including ocular disorders, cancer, cardiovascular, autoimmune, and infectious diseases. Quirico et al. (2020) developed an aptamer-based therapeutic tool for the inhibition of pro-metastatic miR-214 and reduction of metastasis by simultaneously overexpressing its downstream molecule, anti-metastatic miR-148b [70]. The research group designed a chimeric RNA aptamer against the Axl, an oncogenic tyrosine kinase receptor, which is overexpressed by most cancer cells. The aptamer Axl-148b displays high specificity to Axl positive cancer cells, ultimately inhibiting its migration, thus, inducing the apoptosis. Furthermore, the aptamer also blocked tumor cell dissemination in mice, providing a new insight for miRNA-based therapeutic development. In a similar study, Jin et al. (2019) developed an RNA aptamer (HA28) towards chymase, which plays an essential role in the regulation of Ang II in cardiovascular tissues [71]. The effect of HA28 aptamer was evaluated on the cardiovascular tissues, after myocardial infarction. Cardiac parameters such as fractional shortening and left ventricular ejection fraction were improved in the HA28 treated group, significantly reducing the mortality rate, compared to the vehicle-treated hamsters (8%; *p* < 0.05, vehicle versus HA28). This suggested that the application of RNA aptamers for chymase might be a new therapeutic target in post-myocardial disorders. In cancers, hypoxia-induced secreted proteins play an essential role in promoting angiogenesis, wherein the VEGF-A binds and activates VEGFR-1 and VEGFR-2. Yoshitomi et al. (2020), selected two DNA aptamers from 70-mer single-stranded oligodeoxynucleotide libraries, namely Apt01 and Apt02, against VEGFR-1 and VEGFR-2, respectively [72]. Circular dichroism spectra and UV melting curve confirmed that the aptamer Apt01 possesses the stem-loop structure, and other aptamer Apt02 have a G-quadruplex structure. Due to the difference in binding and nuclease stability, the aptamer Apt02 stimulated the tube formation of human umbilical vein endothelial cells. From the results, it could be inferred that Apt02 could function as an alternative to VEGF-A. Carvalho et al. (2019) developed an AS1411(26 nt long DNA aptamer) targeted delivery of acridine-based G-quadruplex ligand (C_8_) to cervical cancer cells. The flow cytometry data suggest that AS1411-C8 complexation have high binding strength, it also significantly decreased the ligands cytotoxicity towards non-cancerous cells. The author also demonstrated the repression of c-MYC expression at the transcriptional level. The reason was possibly due to the ability of C8 to stabilize the c-MYC promoter G-quadruplexes [54]. The schematics of the AS1411-C8 internalization is depicted in Figure 2A.

Bispecific aptamers act as a modulator to bind both, the target receptor and paired protein, simultaneously, when they are in proximity to the cell membrane. Wang et al. (2019) developed a bispecific aptamer, where the paired protein acted as a cancer biomarker and inhibited the function of target receptors via substantial steric hindrance [73]. The application of bispecific aptamers can modulate receptor function and have several therapeutic features for biomedical application. A schematic illustration of the process through which bispecific aptamers regulate Met receptor function are presented in Figure 2B.

The Noxxon Pharma, Berlin, Germany, developed an RNA aptamer, NOX-A12, which is the mirror image of naturally occurring D-RNA molecules (Spiegelmers) [74]. The NOX-A12 is an antagonist of CXCL12 that binds to the chemokine and disrupts the accumulation of chronic lymphocytic leukemia (CLL) cells in the bone marrow [75]. Further, phase I clinical trials for the aptamer NOX-A12 demonstrated a considerable safety profile, when injected into healthy individuals. Recently, the phase II clinical trial of NOX-A12 along with drug bendamustine and rituximab is under progress in patients with relapsed CLL. Similarly, other drugs such as bortezomib and dexamethasone with NOX-A12 are also under clinical trial for patients with relapsed multiple myeloma. Apart from this, several aptamers were also developed to target human epidermal growth factor 2. These were used to deliver therapeutic drugs, RNA, and nanoparticles into the cancer cells [76,77].

Apart from the antagonistic aptamers, several RNA aptamers were designed to act as receptor agonists for improved cancer therapy. To date, limited aptamers functioning as therapeutic agonists have been developed and clinically utilized. These aptamers usually target the co-receptors, namely, CD28, CD40, and 4-1BB (CD137) of the immune signaling pathway [78]. The first-generation agonistic RNA aptamer (K_D_ of 40 nM) was isolated using SELEX against the extracellular domain of murine 4-1BB. The introduction of 2′-fluoropyrimidines in the library as well as in the subsequent pools, conferred the nuclease stability of the RNA aptamer. The bivalent form of the 4-1BB aptamers was generated transcriptionally by streptavidin-coated beads and biotin at 5’end. The bivalent aptamers, as compared to its monomeric form, were able to co-stimulate the T-cell activation, leading to tumor regression in mice models [79]. In a similar line of investigation, two RNA aptamers (CD28apt2; K_D_ = 40nM and CD28apt7; K_D_ = 60 nM) against murine CD28 were isolated from a 2’-fluoropyrimidine-modified RNA library, demonstrating distinct behaviors after the dimerization [80]. The CD28apt2 aptamer blocked the interaction of CD28 with its ligand B7.2. The other aptamer, CD28apt7, was inactive. However, when the two aptamers, i.e., CD28apt2 and CD28apt7, were engineered at 3’ end, they could dimerize with each other and convert into a bivalent form, offering an artificial co-stimulatory signal. The experimental results demonstrated that the bivalent CD28 aptamer enhanced the anti-tumor immune response in the murine lymphoma model and considerably increased the survival rate (*P* = 0.0356 compared with the scrambled aptamer). It can be concluded that aptamers can be engineered to function as antagonistic (blocking) or agonistic (activating) at different times, by employing simple dimerization techniques. However, the counterpart of the aptamers, i.e., antibodies, can act either as antagonistic or agonist.

From the literature, it is evident that the research on DNA and RNA aptamer is rapidly progressing. However, the development of clinically useful aptamer therapeutics is growing slowly, as compared to antibodies. This might be due to the competition between the aptamers and the monoclonal antibody-based drugs. The first FDA-approved aptamer-based drug Pegaptanib (Macugen) targeted against VEGF [81] was also suppressed by VEGF-specific monoclonal antibody based drugs, i.e., bevacizumab (Avastin; Genentech) and ranibizumab (Lucentis; Genentech) [82]. Several other critical factors, such as the lack of knowledge concerning the nucleic acid chemistry, suitable studies on pharmacokinetics and biodistribution of aptamers, production costs, and the reluctance to deter from conventional therapies, did not permit the widespread use of aptamers. The termination of the Phase III clinical trial of factor IXa aptamer has raised concerns amongst researchers, towards the extensive use of aptamers for therapeutic applications [83,84]. On the other hand, numerous start-ups and small-scale enterprisers developed several therapeutic and diagnostic aptamers that have an immense potential. Top aptamer companies, such as Aptamer Group, Optech Biotech, Aptagen, LLC, and Aptamer science have significantly influenced the biotechnology market. SomaLogic Inc. has now generated SOMAmers for more than thousands of crucial proteins that are responsible for various diseases. Further, they have also developed Spiegelmers, SOMA scan, and Soma panel for proteomics and diagnostic applications. However, the clinical development of aptamers is not yet fully achieved, due to suboptimal designs or formulations, base modification, biodistribution, and pharmacokinetics. Despite these limitations, aptamer development is progressing steadily, and the advancement in bioconjugation strategy, nucleic acid chemistry, has encouraged global researchers to pursue therapeutic aptamers for various diseases. 

The Global biotechnology market of aptamers is expected to get flourished from USD176.86 million to an estimated value of USD 722.69 million, registering a CAGR of 19.01% for the period from 2019 to 2026. This sudden increase in market value can be attributed to the increasing R&D investments in the biotechnology and pharmaceutical sectors. In 2004, the FDA approved the therapeutic use of Pegaptanib (Macugen) against all isoforms of human VEGF, and it is currently marketed by Pfizer and Eyetech. Pegaptanib was selected from the 2′-ribopurine/2′-fluoropyrimidine (rRfy) transcript library using SELEX and finally truncated to 27-nucleotide long, to reduce the synthesis cost. To increase the nuclease resistance, 12 out of 14 ribopurines were replaced with 2′-*O*-methyl purines and capped at the 3’-terminus, to decrease the 3′-exonuclease-mediated degradation. The final oligonucleotides were then conjugated to 40 kDa PEG, to increase the tissue distribution and pharmacokinetic inside the cell. The pegaptanib binds to the VEGF and inhibits the interaction with its receptor VEGFR-1 and VEGFR-2, with an IC_50_ value of 49 pM [85]. Similarly, another aptamer ARC1905 by Opththotech was developed to bind the complement component 5 (C5) and was used for the treatment of age-related macular degeneration (AMD). The aptamer selected from 2′-ribopurine/2′-fluoro pyrimidine transcript library using SELEX is currently under a phase I clinical trial and was co-administrated with a VEGF-specific antibody fragment (ranibizumab) [86]. Recently, there is a growing interest in developing aptamers that target cancer-cell surface proteins and biomarkers and further translate them into clinical trials. The first DNA aptamer for cancer treatment was AS1411, targeted for the nucleolin protein. The phase I clinical trial of AS1411 with the patient showing advanced solid tumors yielded significantly good results without any side effects. The phase II trials were also conducted with patients with metastatic refractory renal cell carcinoma. However, the results suggested that the drug had limited activity, indicating the need for specific biomarkers for AS1411 responsive tumors (NCT00881244 and NCT00740441). 

The second successful therapeutic aptamer, i.e., NOX-A12, was an antagonist of the chemokine known as CXCL12/SDF1. The preclinical evaluation indicated that the NOX-A12 activates WBCs, and hematopoietic stem cells in the peripheral blood of mice and healthy human volunteers [87]. Several DNA and RNA aptamers were also developed for blood coagulation and angiogenesis cascade. Platelet-derived growth factor (PDGF) is a mitogen that plays a vital role in the process of angiogenesis, during the early stages of retinal vasculature. To prevent ocular vascular disease, different aptamer-based therapeutic strategies were adopted to block the PDGF activity. Green et al. (1996), for the first time, selected the inhibitory DNA aptamer for PDGF B-chain. The individual DNA ligand clones from the affinity-selected pool bind to the PDGF-AB and PDGF-BB, with a K_D_ of around 10^−10^ M) [88]. The most recent aptamer Fovista, commonly known as Pegpleranib (formerly E10030), which target PDGF, is undergoing simultaneous phase 2/3 clinical trials. Fovista was modified with 2’F-pyrimidines and 2’OMe purines, with an affinity of ~100 pmol/L and a serum half-life up to ~8 h [89]. The in vitro and in vivo studies demonstrated that the aptamer binds to PDGF and blocks its binding to PDGFR-B, preventing the formation of retinal neovascularization. The Phase I clinical trial of Fovista (Formerly E10030) examined the safety, efficacy, and tolerability of human volunteers, in combination with ranibizumab or as a monotherapy. The dose-escalation used was 0.03, 0.3, 1.5, and 3.0 mg, and all doses were well-tolerated without any significant side effects. The phase II randomized study with 449 patients from neovascular AMD also provided promising results, after the use of Fovista. The results depicted a 62% improvement in vision, as compared to the group treated with ranibizumab alone (NCT01089517). However, the aptamer was projected to work synergistically with Macugen or Avastin, for the treatment of age-related macular degeneration (MD) [90].

Several aptamers are under development and have undergone clinical trials related to blood clotting and thrombosis. The von Willebrand factor (VWF), a multimeric glycoprotein, plays a vital role during platelet thrombus formation. The aptamer ARC1779 (49-nt long conjugated with PEG) binds to the A1 domain of the von Willebrand factor (VWF), for use in the acute coronary syndromes. The aptamer ARC 1779 blocks the binding of the A1 domain to the GPib receptor and stimulates platelet activation and adhesion. The randomized placebo-controlled study was performed, and it was found that there were no adverse effects with 47 healthy patients (0.05 to 1.0 mg/kg) [91]. In type 2B von Wilebrand disease, the aptamer ultimately binds and blocks the VWFA1 domain, preventing the drop in platelet amount in patients [92]. Similarly, NU172, a 26-nucleotide long DNA aptamer, was selected, which interfered with the thrombin function. The NU172 was evaluated in phase II for heart diseases by ARCA pharma (NCT00808964). However, there are no recent reports about the completion of this clinical trial. A novel two-component aptamer-based anti-coagulant system, Regado 1a (REG) was clinically evaluated for acute coronary syndrome (NCT00932100 and NCT00715455). It consisted of RB006 and RB007 (complimentary reversal reagent). The aptamer RB006, a PEGylated 31-nucleotide RNA aptamer, was modified with 2- fluoropyrimidine and inverted terminal deoxythymidine residue (IDT) [93] and selectively bound to the coagulation factor IXa. However, the antidote RB007, a 15- nucleotide long unmodified aptamer, bound and neutralized the anticoagulant activity of RB006. The drug RB006 and antidote RB007 were administered to 85 healthy people at random, and it was found that the drugs were well-tolerated without adverse effects [94]. The REG1 system was beneficial for patients undergoing acute coronary syndrome or coronary intervention. A list of the nucleic acid aptamers that are in various clinical stages of development or are being approved, is presented in Table 3.

## 5. Challenges

The application of mono/polyclonal antibodies is the workhorse in clinical practices. However, a major pitfall of antibodies is batch-to-batch variation. ELISA is the commonly employed diagnostic methods for the detection of biomarkers from body fluids for various infectious diseases. The major limitation of ELISA is the multiplexing ability, which can be addressed by the aptasensor. ELISA diagnostic protocols are found to be more sensitive when aptamers are used as recognition elements instead of antibodies. Furthermore, aptamers can be selected against non-immunogenic and toxic targets, whereas antibodies cannot be developed. However, a limited number of aptasensor are in the pipeline for the detection of biomarkers and other microorganisms. The significant limitations associated with the aptamer-based diagnostics is the cross-reactivity or the specificity. The aptamers intended for a selected target could also bind to a closely related or structurally similar molecule. Gening et al. (2006) developed four aptamers against DNA polymerase β, and it showed these aptamers can also bind to the DNA polymerase κ, from another enzyme family [108]. This type of cross-reactivity issue can be addressed by the introduction of negative selection, during the SELEX process. Therefore, a more stringent SELEX procedure is essential for the generation of highly specific aptamer, which can be used for diagnostic purposes in clinical settings. Another type of challenge for the generation of diagnostic aptamer involves the purified target protein. Usually, these proteins are recombinantly expressed in cell culture or prokaryotic cells and are affinity purified using the chromatographic system. However, some proteins are difficult to express and purify. Therefore, the aptamers generated against the target proteins expressed in prokaryotic cells do not interact with the same proteins expressed in eukaryotic cells. This is due to the post-translational modifications inside the eukaryotic cells, and hence the proteins are not able to bind to the aptamers with the same affinity [109]. Therefore, it is necessary to understand the detailed background of the target protein before performing the SELEX. Considering the widespread application of aptamers in biosensors and diagnostic reagents, it is predicted that aptasensor technology is one of the fastest-growing biotechnology areas in diagnostics, with an expectation of reaching about $250 million by 2020 [110].

The last few decades witnessed several oligonucleotides entering into the clinical trials and reaching to the commercial stage. The first FDA-approved oligonucleotide-based therapeutic agent was an antisense oligonucleotide (ASO) named Fomivirsen, used for the treatment of cytomegalovirus retinitis [111]. Following the success, the FDA approved many more oligo-based drugs such as Kynamro, for the management of familial hypercholesterolemia [112], Eteplirsen for Duchenne muscular dystrophy [113], and Spinraza for spinal muscular atrophy [114] were commercialized. Several aptamer-based drugs underwent clinical trials with thousands of participants considering the advent of high-throughput SELEX procedure, nucleotide modification chemistries, and sequencing techniques. However, despite the high potential, only Macugen was approved for the final clinical application by the FDA. The widespread use of therapeutic aptamers is hindered by their inherent physicochemical properties, which affect the metabolic instability, renal filtration, and non-specific immune activation. Therefore, various chemical modifications were adopted to improve the pharmacokinetic properties of aptamer-based therapeutics. However, only certain modifications such as 2’-amino pyrimidines, 2’-fluoropyrimidines, 2’-*O*-methyl nucleotides were successfully integrated into the SELEX procedure. In post-SELEX strategies, modification of aptamer 2’-position was done by introducing a sugar ring or phosphate group, during the solid-phase chemical synthesis. It is worth mentioning that the sensitivity and specificity of the aptamer depend on the secondary structure because of the hydrogen bonds. Therefore, the modification should be tailored accordingly. There is no universal rule available for aptamer modification, but laborious evaluation and optimization is the key to success.

The issue of toxicity is another challenge, although minor, in the clinical evaluation and, therefore, in the success of aptamer-based therapeutics. The potential toxicities might arise due to the polyanionic effects, intensive chemical modification, or undesired tissue accumulation. The toxicological information on aptamers in the human trial is very limited. These toxicological effects might arise due to post-SELEX modifications. The aptamer-based REG1 system developed by Regado Bioscience has raised safety concerns about the use of PEG in the patient in phase III clinical study [115], despite the fact that PEG was approved beforehand. Therefore, accurate therapeutic formulations and a proper administration route is needed for the development of aptamer-based therapeutics. The average size of the aptamers is approximately less than 5 nm in diameter. To increase the circulation time and to overcome the renal filtration, aptamers are generally modified with large groups such as PEG, cholesterol, proteins, and liposomes. PEG is most commonly used and approved by FDA for prolong circulation and half-life; this improves the in vivo bioavailability of therapeutics aptamers [116]. The FDA approved drug, Macugen increased the half-life from 9.3 to 12 hours in plasma, after intravenous or subcutaneous injection, respectively, after PEG modification. The literature suggests that a multivalent aptamer improved the overall performance of binding affinity, specificity, circulation time, and pharmacokinetic properties [117]. The diversity of random oligonucleotide library can exceed the variety of antibodies in the mammalian genome, by several orders of magnitude. In this context, numerous aptamers were already developed as an excellent drug candidate for a wide range of diseases. Several aptamers and aptamers combined with drugs, were progressed into the clinical trial and failed to succeed. The reason might be due to improper chemical modification, biodistribution, and poor pharmacokinetics. In Table 4, we outlined some aptamer-based drugs, which are terminated or withdrawn from the clinical trial studies.

The production of high-purity human proteins that can be used in SELEX, is a complicated process that could be another limiting factor for utilizing aptamers for diagnostic purposes. Additionally, SELEX cannot be used for the identification of unknown targets, proteins that work only in a multiprotein complex, and insoluble proteins [25]. The potential of SELEX for diagnostics is enormous, despite some limitations, such as the complexity of some cancer cell lines and conditions. As cell death results in altered protein expression, such cells must be eliminated for identifying specific aptamers during SELEX. Additionally, the heterogeneity and complexity of some cancer cells require additional rounds of negative selection against non-target cells, to improve the specificity of aptamers [25,118]. Therefore, efforts were made to improve the aptamer selection process, by modifying the SELEX procedure, as mentioned before.

## 6. Conclusions

Aptamer technology remains in a nascent stage, as compared to antibodies, which require further development to ensure its widespread use. Emphasis should be given to the recognition of small molecule targets or targets that are toxic to the cell. The use of next-generation high-throughput sequencing, robust nucleic acid chemistry, would have an essential and lasting effect on the aptamer field. From the literature survey, it can be inferred that the therapeutic aptamers seem to have progressed slower than their application in the analytical and diagnostic areas. Despite several clinical trials, only the Macugen aptamer was approved by the FDA, for its application in clinical use. It is a fact that novel drug molecules take several years of extensive research, before reaching the patients. Recently, the patent of SELEX technology expired, and therefore, as expected, many aptamer-based drugs will be developed for clinical use. Further, to enhance the widespread use of aptamers, the pharmacokinetic properties and biodistribution part of the aptamers should be thoroughly understood. Due to the unique three-dimensional structure of aptamers, they can bind to the target epitopes with high specificity, which is not accessible to the larger antibodies. These advantages of aptamers might ultimately lead to effective fighting with a bacterial and viral infection, cancer immunotherapy, and targeted delivery. Therefore, for the delivery of drug-loaded nanoparticles, aptamers play a vital role in future personalized medicines. Additionally, many aptamers help in internalizing into the cells after binding to the cellular receptors or other components. It can open new avenues for increasing the target specificity and pharmacokinetic profile in the drug delivery system. Right now, we lack in vivo studies, and we hope the approval of aptamer-based medicine in the past and ongoing clinical trials will open new horizons.

## Figures and Tables

**Table 1:** A list of aptamers in the literature used for diagnosis of cancer cell lines through in vivo and in vitro SELEX.

Target	Aptamer Sequence (5’-3’)	SELEX Method	Binding Affinity	Brief Result	Ref
MCF-7 breast cancer cells	GGTGGTGGTGGTT-GTGGTGGTGGTGG	Cell-SELEX	30–50 nM	**AS1411** Aptamer-AuNP probes for color-based visual detection of MCF-7 breast cancer cells with a detection limit of 10 cells.	[38]
Prostate cancer cells	GGAGGCAACGGAG-CGGAGACATTGAC-TGAGTGAACGTGT-AGTG	In vivo SELEX	2–100 nM	**D3-21** aptamer conjugated with PEG was used for in vivo detection of prostate cancer by in vivo SELEX	[39]
Blood cancer or leukemia cells	TTTTTTTTTTATCT-AACTGCTGCGCCG-CCGGGAAAATACT-GTACGGTTAGA	Live cell-SELEX	-	Amine-labeled **Sgc8** aptamers were conjugated to carboxyl-modified fluorescent silica NPs for highly specific and sensitive detection of leukemia cells.	[40]
Leukemia cells	TTTTTTTTTTATCT-AACTGCTGCGCCG-CCGGGAAAATACT-GTACGGTTAGA	Live cell-SELEX	5.16 nM	**Sgc8** aptamers were conjugated to AuNP-coated magnetic Fe_3_O_4_ NPs for highly specific and sensitive detection of leukemia cells.	[41,58]
HepG2 liver cancer cells	ACAGCATCCCCAT-GTGAACAATCGCA-TTGTGATTGTTAC-GGTTTCCGCCTCA-TGGACGTGCTG	Live cell-SELEX	-	**TLS11a** aptamer-fluorescent silica NPs conjugates for detection of liver cancer cells, HepG2.	[42]
Nucleolin & Tenascin-C cancer biomarkers	TTGGTGGTGGTGG-TTGTGGTGGTGGT-GG & CCTGCACTTGGCT-TGGATTTCAGAAG-GGAGACCC	Cell SELEX	-	**AS1411** and **TTA-1** aptamer-conjugated with QDs were used for multiplex detection of nucleolin and tenascin-C cancer biomarkers.	[43]
Glioma cancer cells	GCAATGGTACGGT-ACTTCCTGAATGT-TGTTTTTTCTCTT-TTCTATAGTACAA-AAGTGCACGCTAC-TTTGCTAA	Cell SELEX	-	**A32**-aptamer-QD conjugates were used for fluorescence-guided surgery for glioma cancer cells	[45]
Ovarian cancer Cells	TCTCTAGTTATTG-AGTTTTCTTTTAT-GGGTGGGTGGGG-GGTTTTT	Cell SELEX	29.24–158 nM	**R13** aptamer showed a high binding affinity with several ovarian cancer cell lines (Caov3, HO8910, A2780, and SKOV3)	[47]
Gastric cancer cells	GGATCCGACACGA-CCCTATAGTGAGT-CGTATTA	Cell SELEX	16.5–156, 52.7–71.2, 30.7–38 nM	Aptamers with high affinity against gastric cancer biomarkers (CEA, CA72-4, and CA50) were selected.	[48]
Gastric cancer cells	CCTCGGCACGTTC-TCAGTAGCGCTCG-CTGGTCATCCCAC-A	Whole-serum subtractive SELEX	128 nM	Highly specific aptamer (**Seq-3**) for gastric cancer was selected through the whole-serum subtractive SELEX	[12]
MCF-7 and 4T1 breast cancer cells	GGTGGTGGTGGTT-GTGGTGGTGGTGG	Cell SELEX	30–50 nM	Fluorescein-labeled **AS1411** Aptamers were integrated with an ultrasound-propelled gold nanowire motors (FAM-AIB1-apt) and MRI machine for qualitative diagnosis of breast cancer cells.	[50,53]
Leukemia cells	ATCTAACTGCTGC-GCCGCCGGGAAAA-TACTGTACGGTTA-GATTTTTTTTTT	Cell SELEX	0.04 Hz	**Sgc8** aptamers were integrated into a microfluidic device for rapid detection of leukemia cells.	[51]
HepG2 liver cancer cells	ACAGCATCCCCAT-GTGAACAATCGCA-TTGTGATTGTTAC-GGTTTCCGCCTCA-TGGACGTGCTG	Cell SELEX	-	**TLS11a** aptamer was conjugated with Fe_3_O_4_ NPs for rapid and specific detection and bio-imaging of HepG2 liver cancer cells in combination with MRI.	[52]
AML cancer cells	TGCGTGTGTAGTG-TGTCTGTTGTTTG-TATTGTTGTCTAT-CCTCTTAGGGATT-TGGGCGG	In vitro SELEX	101 nM	**AB3** aptamer was functionalized to deliver doxorubicin (Dox) drug molecules to the acute myeloid leukemia (AML) cancer cells.	[57]

**Table 2:** A list of aptamers in the literature applied for the detection of infectious pathogens.

Target	Aptamer Sequence (5’-3’)	SELEX Method	Binding Affinity	Brief Result/Specific Nanoparticles	References
*Candida albicans*	-	Cell SELEX	79.76 nm 103.7 nM	**AU1** and **AD1** aptamers were used for the detection of the (1→3)-β-d-glucans present in the cell wall of the fungus, *Candida albicans* with high affinity and specificity.	[38]
*Escherichia coli* ATCC 8739	GCAATGGTACGGT-ACTTCCCCATGAG-TGTTGTGAAATGT-TGGGACACTAGGT-GGCATAGAGCCGC-AAAAGTGCACGCT-ACTTTGCTAA	Cell SELEX	-	The FRET aptasensor detected E. coli ATCC 8739 with a LOD of 3 CFU/mL	
Dengue virus 2 (DENV)	GCACCGGGCAGGA-CGTCCGGGGTCCT-CGGGGGGC	In vitro SELEX	200 nm	Aptamer **S15** with high affinity and specific diagnosis of the envelope protein domain III (ED3) of dengue virus 2 (DENV).	[64]
Influenza viruses (H3N2 and H1N1)	-	Subtractive SELEX	5.56–5.84 nM	**A8** and **A20** DNA aptamers and their truncated sequences used for detection of type A influenza viruses (H3N2 and H1N1 viruses) with high affinity and selectivity	[11]
Ebola virus & Ebola Sudan virus	GGGCGCUCAAUUU-UUUAUUGCAUUUU-UCUUUGAGCGCCC	Cell SELEX	30 nM & 250 nM	An RNA aptamer, 39SGP1A, functionalized with 2’ fluoropyrimidine (2’FY) for efficient detection of Ebola virus (EBOV) and Ebola Sudan virus (SUDV).	[65]
Chikungunya & Zika viruses	-	Cell SELEX	50 pg/mL	An Aptamer-Au NPs conjugate based device for multiplexed colorimetric diagnosis of chikungunya and Zika viruses with high selectivity in a microfluidic channel. The subsequent introduction of silver reagent and its deposition on the AuNPs surface created a gray contrast in the testing zone.	[66]

**Table 3:** Nucleic acid aptamers currently in the clinic.

Aptamer	Modification	Target and Binding K_D_	Application	Clinical Status	Ref.
Pegaptanib sodium (Macugen) RNA (27 nt)	2’fluoropyrimidines 2’-*O*-methyl purines 3’-inverted dT 40 kDa PEG	VEGF_165_ 50 pM	AMD Diabetic macular edema Diabetic retinopathy	FDA approved drug for the treatment of AMD	[95,96]
ARC1905 (Zimura) RNA (38 nt)	2’fluoropyrimidines 2’-*O*-methyl purines 3’-inverted T 40 kDa PEG	C5 20-40 nM	Dry AMD IPCV	Phase I completed, Phase II and III recruiting (NCT02686658) Zimura in Combination with Anti-VEGF Therapy in Subjects with IPCV (NCT02397954)	[86]
E-10030 (Fovista) DNA (29 nt)	2’-*O*-methyl purines 3’-inverted dT 40kDaPEG	PDGF 20 pM	Neovascular AMD	Phase II (NCT02214628) Anti-PDGF Pegylated Aptamer with Lucentis (NCT01089517) for neovascular AMD Fovista in Combination with Lucentis as compared to Lucentis monotherapy (NCT01940900)	[97]
NOX-A12 RNA (45 nt)	PEGlyated L-RNA (Spiegelmer)	CXCL12 200 pM	CCL Multiple myeloma Colorectal cancer Pancreatic cancer	Phase II (NCT01486797) NOX-A12 in Combination with Bortezomib and Dexamethasone Phase II (NCT 01521533)	[75]
AS1411 DNA (26 nt)	G-rich quartets, PEGlyated	Nucleolin 55 nM	AML MRCC	Phase II (NCT01034410) Phase II (NCT00740441) Phase I (NCT00881244)	[98,99]
NOX-H94 (lexaptepid pegol) RNA (44 nt)	L-RNA 5’ with 40 kDa PEG	Human Hepcidin 0.65 ± 0.06 nmol/L	Anemia of chronic disease End-Stage Renal Disease	Phase I and II (NCT02079896)	[100]
68Ga-Sgc8 DNA (41 nt)	Bifunctional agent 1,4,7-triazacyclononane-1,4,7-triacetic acid (NOTA) Radioisotope Ga^68^	PTK7/ CCk-4 NA	Colorectal cancer	Early Phase 1 (NCT03385148)	
NOX-E36 RNA (40 nt)	L-RNA, PEGlyated	MCP-1 1.32 nM	Chronic Inflammatory Diseases Type 2 Diabetes Mellitus Systemic Lupus Erythematosus	Phase I (NCT00976729)	[101,102]
NU172 DNA (26 nt)	G-quadruplex structure and unmodified	Thrombin 0.3–0.5 nM	Heart disease	Phase II (NCT00808964)	[103]
ARC1779 DNA (39 nt)	3′-inverted dT 2’-*O*-methyl group 20 kDa PEGlyated	Von Willebrand factor (A1 domain) 2 nM	Purpura, Thrombotic Thrombocytopenic von Willebrand Disease Type-2b Acute Myocardial Infarction	Phase II (NCT00632242) Phase II (NCT00507338)	[104,105]
REG1 anticoagulation system (RB006 and RB007) RNA (37 nt) (RB006) Antidote (RB007)	2’-ribo purine or 2’fluoropyrimidine 40kDaPEG	Coagulation factor IXa NA	Acute coronary syndrome Coronary artery disease Percutaneous coronary intervention	Phase I and II completed (NCT00113997, NCT00932100, NCT01872572)	[106,107]

VEGF: Vascular endothelial growth factor; AMD: Age-related macular degeneration; IPCV: Idiopathic polypoidal choroidal vasculopathy; C5: Complement 5; PDGF: Platelets derived growth factor; PTK7: Protein tyrosine kinase 7; CCK-4: Colon carcinoma kinase-4; PEG: Polyethylene Glycol; MCP-1: Monocyte Chemoattractant Protein 1 CXCL12: Chemokine ligand 12; CCL: Chronic Lymphocytic Leukemia; AML: Acute Myeloid Leukemia; MRCC: Metastatic renal cell carcinoma; and K_D_: Kinetic binding constant.

**Table 4:** Nucleic acid aptamers removed from the clinical trials.

Drug Candidates	Targeted Disease	Clinical Phase	Result	Company
Fovista^®^ (anti-PDGF BB) plus anti-VEGF	Age-related Macular Degeneration	Phase II (NCT02214628)	Terminated	Ophthotech Corporation
Drug: E10030 Drug: ranibizumab Drug: E10030 sham intravitreal injection	Age-related Macular Degeneration	Phase III (NCT01944839)	Terminated	Ophthotech Corporation
Drug: E10030 Drug: bevacizumab or aflibercept Drug: E10030 sham intravitreal injection	Age-related Macular Degeneration	Phase III (NCT01940887)	Terminated	Ophthotech Corporation
Drug: Pegaptanib sodium	Macular Degeneration	Phase IV (NCT00312351)	Terminated	Eyetech Pharmaceuticals
Drug: ARC1779	Von Willebrand Disease	Phase II (NCT00694785)	Withdrawn	Archemix Corp
Drug: placebo control Drug: ARC19499	Hemophilia	Phase I (NCT01191372)	Terminated	Baxalta Inc
Drug: AS1411Drug: Cytarabine	Acute Myeloid Leukemia	Phase II (NCT01034410)	Terminated	Antisoma Research
